# *Making Room to Be OK*: A Feminist Grounded Theory Study of How Women Manage Their Health in the Context of Suicide Ideation

**DOI:** 10.1177/23333936261442946

**Published:** 2026-04-27

**Authors:** Petrea Taylor, Sue O’Donnell, Kelly Scott-Storey, Jeannie Malcom, Charlene Vincent

**Affiliations:** 1 University of New Brunswick, Moncton, Canada; 2 University of New Brunswick, Fredericton, Canada

**Keywords:** suicide, women’s health, mental health, grounded theory, feminism, borderline personality disorder, Canada

## Abstract

Suicidal ideation (SI) is interpreted through a biomedical lens across health care systems, framing it as a pathology requiring treatment and leading to approaches aimed at controlling or eliminating these thoughts. Within this dominant medical model, illness treatment and death prevention are prioritized, eclipsing attention to health and well-being. Our previous research shows that women make efforts to improve their health despite living with SI. Using a feminist grounded theory approach, we sought to understand how women with SI manage their health. Data from interviews with 32 Canadian women were analyzed using the constant comparative method, elevating the data to a higher level of abstraction. We found that women with SI manage health by *making room to be OK*, creating space within their environments that allows them to better manage unbearable psychological pain. *Making room to be OK* becomes possible through *acceptance*, social recognition that ending unbearable psychological pain is a legitimate health need. Approaches critical to helping women *make room to be OK* include offering spaces within healthcare and community settings where SI can be discussed without pressure to think or feel otherwise. These trauma- and violence-informed approaches diverge from dominant medical services that seek to control women’s suicidality.

## Introduction

Suicidal ideation (SI) is highly misunderstood as a pathology requiring treatment with little consideration given to health and well-being while living with SI. Most literature on suicide focuses on suicide death and risk factors with less knowledge on SI and the experiences of living with SI (Gay, 2025a). Although there have been strong critiques of the dominant medical model’s focus on suicidality as pathology, the focus of the literature is the treatment of suicidality, making death prevention a priority in comparison to health improvement and quality of life (Krebs, 2025; Marzetti et al., 2025; Ward, 2026). The medical model contributes to the following binary assumptions about suicidality: either a person (a) has SI, has a high risk of dying, and is uninterested in health or (b) does not have SI, has a low risk of dying, and is committed to health. SI and health, however, are not dichotomous variables; rather SI is situated in ambivalence: the wish to end psychological pain through suicide and to live or to be well can occur simultaneously (Galasiński & Ziółkowska, 2024).

A risk of these binary assumptions is that in the absence of a highly lethal suicide plan or attempt, people with SI are not taken seriously (Maple et al., 2020). SI can exist for months or years (Tsypes et al., 2024) with little consideration from health care providers about the role of health and wellness. Health care providers’ minimization of SI and non-lethal suicide attempts may disproportionately affect women because they are more likely to attempt, plan, and think about suicide in comparison to men (Public Health Agency of Canada, 2023). Minimizing women’s experiences of suicidality is evidenced by the disproportionate and often inaccurate labelling of women with borderline personality disorder, a highly stigmatized psychiatric condition characterized by emotional dysregulation, chaotic relationships, and chronic suicidality (American Psychological Association, 2010). Borderline personality disorder (Day et al., 2018) or women with ongoing SI (Taylor, 2022a) is documented as a sign that health care providers interpret women with SI as being attention-seeking, while women’s past and ongoing trauma and violence experiences are ignored (Brown, 2024). In a previous study, the help-seeking goal for women living with SI was not to end SI, but rather to get help for other health problems including feelings of depression or anxiety as well as for relationship difficulties (Taylor, 2020a). Despite thoughts of ending their lives, women make efforts to promote their health (Taylor, 2020a), but how they do so is not well understood.

Given the narrow focus on suicide pathology, consideration of health and wellness among women living with SI is necessary. Thus, authors sought to address the limited understanding of health behaviours among women in the context of SI. The purpose of this study was to explore how women living with SI manage their health and wellness. The study was guided by feminist grounded theory, an approach used to analyze social processes while remaining attentive to potential power imbalances (Wuest, 1995). Findings will inform policies and clinical practice, offering a strength-based approach to supporting women with SI.

## Literature Review

### The SI Experience

Insight into women’s experience of SI is found within a scoping review of 28 qualitative studies between 1997 and 2021 that examined the experiences of adults with SI (Søndergaard et al., 2022). One notable theme across all studies was the influence of social connections. Research participants with SI experienced interpersonal violence, relationship losses, negative interactions with others and a lack of positive relationships, leading to isolation, disconnectedness and continued SI (Søndergaard et al., 2022). Another striking theme was the loss of a sense of personhood or self-worth, especially when comparing oneself to others which also contributed to SI, whereas a positive self-identity reduced SI (Søndergaard et al., 2022). Since 2022, similar themes were found within qualitative studies about SI, including experiencing social disconnection and negative self-identity among adult psychiatric in-patients (Hagen et al., 2020; Knizek et al., 2024), adolescent and young adult students (Pandya & Raut, 2022), sexual minority university students (Hoskin et al., 2024), and transgender older adults (Gaveras et al., 2023). Additional themes include feeling indifferent and a sense of meaninglessness about life (Galasiński & Ziółkowska, 2024), and having a limited sense of control of one’s life-trajectory (Gaveras et al., 2023).

### Help-Seeking for Suicidal Ideation

Most of the literature on help-seeking for suicidality focuses on frequency of healthcare provider contact, reporting findings related to frequency of contact. For example, most Canadian decedents of suicide had been in contact with a health care provider shortly before their death (Shah et al., 2018) and women are consistently more likely to reach out for help with SI than are men (Public Health Agency of Canada, 2023). Importantly, for those who do seek help for SI, the experience is difficult. Among a mostly female sample of Black university students with SI, only 66% were receiving mental health help (Busby et al., 2019). In a study of firefighters, most women firefighters with SI did not reach out for help due to lack of access to counsellors, professionals with whom they preferred to speak (Hom et al., 2018). Commonly, when people do reach out for help, they do not receive the support they are seeking. Seeking help in the ED has been found to contribute to feelings of abandonment and mistreatment among people in an acute suicidal crisis (Anderson et al., 2024). Access barriers are evidenced by reluctance to seek help due to stigma (Rastogi et al., 2024), especially during periods of higher SI intensity (Blais et al., 2023). Women who had experienced intimate partner violence reported a diminished sense of agency when health professionals’ responses to their help-seeking for SI were perceived as authoritative or judgmental (Taylor, 2020b), which drove them to distance themselves from health services (Taylor, 2022b).

### Treatment of Women with Suicidal Ideation

Whereas the literature on treatments for SI does not typically focus specifically on women, research on interventions for borderline personality disorder provides the most substantial body of treatment evidence specific to women with SI. Dialectical behavioural therapy, a skill-based counselling approach originally created to help manage symptoms of borderline personality disorder through mindfulness-based techniques (Linehan, 1993), is deemed to be the gold standard treatment for women with SI (Austin et al., 2020; Smith & MacDougall, 2020). Although suicide behaviour and self-harm were significantly reduced with dialectical behavioural therapy treatment as demonstrated by 18 randomized controlled trials, SI was not (DeCou et al., 2019). Women with suicidal ideation (SI) and borderline personality disorder define success in terms of meaning, enjoyment, and overall well-being rather than symptom reduction alone (Liljedahl et al., 2023); however, because standard suicidality treatments prioritize symptom reduction (Pemau et al., 2024), they may fail to meet women’s broader health and well-being needs. Further, mainstream treatments for SI narrowly focus on requiring the individual fix their mental health difficulties while ignoring broader issues that contribute to SI and borderline personality disorder symptomology for women including structural violence (Quenneville et al., 2020; Rodriguez-Seijas et al., 2023; Valero et al., 2025). Due to a lack of research on women’s health and wellness in the context of SI, we sought to explore health promotion in women with SI.

### Methods

#### Research Design

Feminist grounded theory (GT) was used for this study. The following are three main versions of grounded theory consistently documented in the literature: (1) classical or Glaserian, (2) Straussian, and (3) constructivist (Rieger, 2019; Singh & Estefan, 2018). Feminist GT is occasionally recognized as the fourth version (Fernandez, 2012; Mohajan & Mohajan, 2022). Judith Wuest first developed feminist GT by applying the Glaserian approach with a feminist lens (Wuest, 1995). Feminist GT is true to classical GT by avoiding verification through forcing data into preset coding paradigms; instead, the analysist promotes emergence through a carefully constructed analysis that allows findings to arise from the data. Feminist principles are addressed in GT by being theoretically sensitive to power imbalances and the role of social factors, including gender, in the data and inviting collaborative knowledge creation with marginalized communities as an approach to generating opportunities for social change (Mohajan & Mohajan, 2022; Wuest, 1995). It was anticipated then that the use of feminist GT would offer a theoretical lens for examining how women with SI manage or promote their health, offering rich accounts of how participants manage health challenges, overcome barriers, and use their strengths to meet their needs.

#### Data Collection

Upon receiving approval from the University of New Brunswick Research Ethics Board (#2020-060), participants were invited to take part through online advertisements. Potential participants were given study details, opportunities to ask questions, and assurance that they could end participation at any time. After providing informed consent, 32 participants shared their experiences. Semi-structured interviews lasted approximately 60 to 90 minutes and participants were prompted using the question, “Please tell me how you manage your health and wellness while living with SI.” The terms health and wellness were participant-defined. The first author has extensive experience as a nursing suicide counsellor, expertise that helped to safely engage participants and provide support during interviews. Demographic information was collected and documented elsewhere (Taylor et al., 2025). Audio renderings from the recorded video interviews were immediately sent for verbatim transcription to a confidential transcription service, upon which time the recording was deleted.

#### Data Analysis

Transcripts were uploaded to NVivo^®^, a qualitative analysis software. Verbatim interviews were coded line-by-line, followed by substantive coding while constantly comparing pieces of data with incoming data and collapsing codes into broader categories. For example, open coding of data that described women’s daily activities and overall functioning in the home were named *creating routines, structured time, checking off lists, one step at a time, one foot in front of the other, functioning in chunks*, and *taking stock*. Theoretical sampling helped to explore these concepts related to routine with subsequent participants by adding an interview prompt, “Tell me about how you think about, frame, organize, or go-about tackling your health.” If women were not sure what this meant, the following additional prompt was given, “One example might be a routine,” which yielded codes about functioning in units of time and space. The codes that survived the constant comparison process collapsed into a category named *creating room or space to function*.

Another category was created to reflect several codes that depicted psychological pain management including *feeling in control, processing pain, identifying my needs, knowing who I am, addressing the here and now, embracing the darkness*, and *feeling my emotions* collapsed into a category named *feeling OK to be me*. The 6 C’s, the family of theoretical codes, was used to determine the cause, condition, contingency, co-variant, context and consequence between categories, raising the data to a higher level of abstraction (Glaser, 1978). *Feeling OK to be me* emerged as the goal of *creating room or space*. This hypothesis was constantly checked against all pieces of data within each interview associated with pain relief, including the code *finding some relief of distressing situations*. Extraneous detail that did not survive the constant comparative process was teased out while emerging data accounted for variation among participants, facilitating the achievement of theoretical saturation, when no new information emerged from the constant comparative analysis (Glaser, 1978), with the basic psychosocial process, *making room to be OK*.

#### Quality and Rigour

The theory within a GT study demonstrates rigour if it has “fit” (Wuest, 2000) or “correspond[s] closely to the data if it is to be applied in daily situations” (Glaser & Strauss, 1967, p. 238). *Making room to be OK*, the basic psychosocial process of health management in women with SI, corresponds closely to the data as it reflects the intended outcome to feel capable of managing pain, rather than seeking to be rid of SI. The theory also must have “grab” or be appealing to the reader, and be judged as it “works,” the ability of the theory to reflect the particulars of what is occurring and to be relevant to the particular area of inquiry (Glaser, 1978). These elements of rigour are reflected in this theory as *making room to be OK* uses every-day language that creates images in the mind regarding how women manage SI in the context of their daily lives. The findings are relevant and valid when the core variables emerge directly from the data (Glaser, 1978), a criterion demonstrated by a detailed audit trail on how theory concepts were derived inductively and deductively tested via an ongoing process of constant comparison (Wuest, 2012). Examples of the audit trail and the construction of findings are demonstrated in the data analysis and findings sections.

The theory of *making room to be OK* also aligns with Glaser’s (1978) concept of “modifiability,” the capacity of the theory to adapt to emerging new dimensions in the data. As data about having few social spaces to talk about SI was becoming more apparent, new data was simultaneously being collected from racialized participants, yielding data about having little space to exist as a person of colour. This deepened our understanding of the role of identity, that living with SI is a part of women’s identity and the capacity to feel better includes others’ *acceptance* of their personhood. These findings may be transferred to other populations experiencing mental health related social stigma. Credibility of the findings was strengthened through the use of a relational approach by demonstrating mutuality with participants and acknowledging power imbalances, bolstering participants’ capacity to express themselves openly (Taylor, 2023).

Finally, the current theory is a contribution to the Glaserian-version of substantive mid-range theories, having met the criteria for rigour and theory building as reflected in the findings being “written at a theoretical level,” as opposed to a descriptive level, and that variations of the core process are identified (Wuest, 2012, p. 248). The current theory raises women’s descriptive accounts of managing health in the context of SI with a basic psycho-social process that represents a diversity of findings and patterns of behaviours with dimensions explained by social context. Variability within the theory dimensions provides social conditions that can be adapted to or amended and used to inform interventions.

## Findings

### Making Room to Be OK

**Figure 1.**
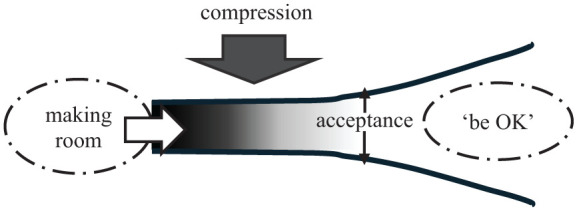
Making room in the social environment to be OK: A health management response to compression.

Women living with SI manage their health through a basic psychosocial process of *making room to be OK*, creating space in their personal environment, community, and society wherein they can better manage unbearable psychological pain. Psychological pain related to past and ongoing trauma and violence, mental or physical health conditions, or other distress is so intense that ending the pain through a variety of socially unacceptable strategies is perceived as a health need. One strategy women reported using to manage pain may involve thinking about ending pain through suicide. The barrier to managing pain is *compression*, the state of being squeezed out of social spaces due to external rejection of SI and disapproval of women’s difficulty in day-to-day functioning. *Compression* is experienced as a heavy mental or emotional weight pushing down on them, condensing their environment, leaving women with reduced room to exist and live authentically (Taylor et al., 2025). *Making room to be OK* is an active and dynamic process of adapting to and resisting the heavy weight of *compression* and is contingent on *acceptance* of women’s health needs. To illustrate the theoretical relationships, we constructed Figure 1.

The action of “*making” room*, located at the far left of Figure 1, is both a conscious and unconscious process of carving out a path and creating segments of space through the weight of *compression* (block arrow top of Figure 1) to address one’s health needs to manage psychological pain by living authentically as a person with SI. A participant demonstrated how she made room by resisting pressures to prioritize others’ needs: “I can’t care for anybody else. I can’t. So I’m just going to care for me. And that’s how I start promoting more of like, I want to go for a walk, I’m going to eat a little bit more.” The participant’s *making room* actions were health promoting behaviours that helped her to take care of herself. The goal of *making room* is to *be OK*, to achieve a sense of comfort and confidence that psychological pain is manageable and to find meaning in the pain. *Being OK* is characterized as approaching pain management with agency and purpose. This is operationalized by being actively and effectively involved in taking control of daily difficulties. In this way, *to be OK* is women’s conceptualization of health. Properties of *being OK* include the capacity to *be me*, to live authentically, to understand one’s health needs, and recognize one’s personal identity and values. Importantly, ending SI is not required to *be OK*, nor is feeling happy or at peace. A participant described *being OK* by explaining that although, “I’m not confident that I will never not think about suicide and dying by suicide, [I’m] fine because I’ve found ways to cope with it.”

*“Room” to be OK* exists within the internal and external environment. *Room* operationalized internally is the perception of time, energy, and space needed to think and be OK. Temporal units are constructed as increments of future orientation measured in seconds, minutes, hours, days, months, years, or the amount of time women have the cognitive capacity to visualize. *Compression* shortens temporal units, decreasing the amount of *room to be OK*. A participant reported that her future orientation was reduced at the beginning of the COVID pandemic that had prevented her from regular in-person visits with her thesis supervisor who provided her with *acceptance* and support: “The only thing I could do [. . .] was [to] talk to [my supervisor. . .] I started having suicidal thoughts [. . .] I had to live day-to-day. [. . .]. I would just get overwhelmed at the thought of basically a future.” Living day-to-day meant that waiting to see her supervisor was easier by existing within smaller blocks of time. She created digestible bits of room that helped her get through *compression* in a step-by-step fashion. When the participant reestablished access to the university community post-emergency lockdown, she was able to continue living in larger blocks of time: “I can confidently say right now that I don’t want to end my life before I finish this degree.”

*Room* is also created by conjuring energy and space to exist in the present. One participant *made room to be OK* using an inner dialogue to get in touch with herself, “OK, my anxiety’s getting high, so which tool am I going to use? Do a body scan, do just breathing, just get up and do yoga.” *Room* operationalized externally is having space within the physical surroundings, such as where women live, health care institutions, or workplaces; social surroundings including the people in women’s lives, and social narratives about mental health including cultural surroundings within families, communities, healthcare institutions and the political climate. Women can *make room to be OK* internally even if there is limited room externally. A participant described *making room* as, “maintaining a clean, healthy environment” by relying on her support system and going out for a walk. *Room* within this wide external environment is operationalized as a sense of belonging or relating to others and the ability to meaningfully engage in the surroundings.

### Acceptance

*Making room to be OK* is contingent on social *acceptance*, external and internal recognition that to end unbearable psychological pain is a health need. In this way, strategies used to reduce psychological pain, including, but not limited to SI and other socially unacceptable behaviours, are also health needs. *Acceptance* is an acknowledgment and embracement of the strategies women use to manage unbearable psychological pain as health needs. *Acceptance* is represented in Figure 1 with thin arrows signifying the capacity to create space in the social environment.

*Acceptance* occurs externally from others or society, internally as women’s *acceptance* of themselves, or both. *Acceptance* reduces *compression*, freeing space and energy needed to manage pain. A participant shared, “I think my biggest want in the world right now is for a, whatever, health professionals to talk about suicide like anything else.” Discussing suicide as a common, informal topic indicates *acceptance* of women’s realities living with SI. External *acceptance* occurs through direct and indirect social messaging, embracing women’s personhood and strengthening their capacity to exist authentically in social spaces wherein they are free to communicate SI and share life problems with others. A powerful form of *acceptance* is being acknowledged and valued for the parts of women’s identity or personhood that make them feel different and disconnected from others and the world around them.

The clearest indicator of external and internal *acceptance* is freedom from the expectation to change. Understanding the need to end unbearable psychological pain supports others’ *acceptance* of SI and other social infractions or pain management strategies that are socially unacceptable. A participant explained that her daughters’ *acceptance* of her self-harm, a coping strategy that she perceived as overlapping with SI as ways of escaping pain, helped to decrease her distress. When the participant’s boyfriend was seeking to control her self-harm by finding and removing razor blades the participant was using to cut herself, the daughters advocated for their mother by explaining how they support her by helping to clean up after she has self-harmed, and told him, “‘Don’t try to take her blades from her. She’ll stop when she’s ready to stop.’ So that made life easier because that was frustrating for me.” The participant was relieved about not feeling pressured to stop cutting. *Acceptance* of self-harm does not equate to promoting self-harm rather, the daughters’ *acceptance* of self-harm reflected in this example highlights understanding of their mother’s need to manage unbearable psychological pain. A participant explained that others’ inability to accept SI “bugs” her, saying, “Accepting the suicidal thoughts [. . .] [is] not necessarily [. . .] a bad thing or a good thing. It’s just something that is.” Although some people in women’s lives had difficulty accepting their SI due to fears of the women dying, they demonstrated *acceptance* to the women when the women perceived that they were not trying to control their SI. A participant explained that “When they don’t freak out when I’m having those suicidal thoughts,” it helps assure her that she can reach out to them for help if needed. Women may stop hiding their SI if they know someone will not argue with them or try to convince them to live.

*Acceptance* from within society includes recognition of differences in abilities within an ableist society that stigmatizes mental illness. A newcomer to Canada reported feeling like an outsider living with depression in her home country where mental illness was not recognized. She gained a sense of freedom and belonging when she learned that workplaces allow sick leave for mental health problems. Another participant described the importance of widespread support within a community, evidenced by organizations that provide help with access to resources. She recounted the following about needing community resources for people with SI: “Community support is often overlooked [. . .], people who connect other people with resources in the community, having community functions, just like a good community structure.” Because *acceptance* includes being recognized for experiencing SI, the lack of this supportive community structure for those with SI highlights the dearth of *acceptance* within community. *Acceptance* is also limited structurally in the community. For example, involuntary apprehension by the police when a person is deemed to be at risk of harm to themselves is the most severe form of *compression* because women perceived their right to freedom was rejected (Taylor et al., 2025). On the contrary, *acceptance* from service providers who enact these laws and policies can counteract *compression*. Although the police came to a participant’s home during an SI crisis to take her to the hospital involuntarily, the police officer’s approach helped to limit *compression*. She explained, “[Officer] came to the door and he said, ‘Bad day [participant’s name]?’ So I started crying. I just broke. ‘It’s just a bad day, [officer’s name].’” “Breaking down” in front of the officer was a sign of feeling safe due to his validating of her distress. Conveying *acceptance* by acknowledging her situation as “having a bad day” made the problem feel more manageable.

Internal *acceptance* or women’s recognition of their need to end unbearable psychological pain helps them address it. Women find ways to reduce their pain to tolerate living and endure the circumstances of the next minutes, hours, days, months, or years. *Acceptance* of SI as a health need reduces efforts to resist it, freeing space to visualize options for pain management. A participant described *acceptance* of her SI,

So it’s kind of been less about, like, Oh well, I’m feeling suicidal so I need to do something about this, because clearly this is the way it is, and kind of just shifting that towards seeing it more as, like, OK, this is, like a day, or maybe a few days, or a week where I feel this way, but it’s not – but, you know, this is just about surviving this part.

Recognizing that SI was time limited made surviving the pain achievable. Among study participants, internal *acceptance* was the most common form of *acceptance* of SI. Indeed, others rarely understood and acknowledged SI as a health need to reduce or escape unbearable psychological pain and an impetus to address it.

### Managing Social Expectations

*Making room to be OK* involves sub-processes: managing social expectations by *meeting expectations* and *permitting myself to be me*.

#### Meeting Expectations

*Meeting expectations* occurs in the context of high *compression* by adopting social pressures to reduce social rejection. This means that women enact social expectations to avoid perceived social judgement by making efforts to be productive and appear they are committed to living. By going through the motions of fulfilling their roles and responsibilities, women are attempting to reduce their sense of *compression* caused by others’ disapproval. *Meeting expectations* involves pretending not to have SI, fighting fatigue, dismissing psychological pain, pushing oneself to be productive at work or school, and fulfilling gender roles including meeting gender role expectations, such as housework, caregiving, and prioritizing the needs of others. The role of *acceptance* in *meeting expectations* is internal acknowledgment of whatever it takes to *be OK*, even if it means distancing from their authentic selves. Although *meeting expectations* minimizes women’s own health needs, by not taking seriously or neglecting their need to rest and explore SI, it functions as a strategy to please others and reduce perceived pressures to conform to social norms. By *meeting expectations* women helped to reduce the weight of external pressure to be different than who they are. In a way, knowing what was expected of them provided a framework within which to manage *compression*.

*Meeting expectations* yields more space to *be OK* than expending significant amounts of energy to resist social expectations, functioning as a path of least resistance effectively reducing the feeling of *compression. Meeting expectations* is an economical way of *making room to be OK* as it avoids making things worse when women are drained and have few resources to fight back. A participant with an eating disorder stopped taking psychotropic medication prescribed for SI due to the side effect of weight gain. She explained, “I was horrified. The cutting got worse, sleep pattern got worse, definitely stopped eating all together. [Dr. name] doesn’t know I’m not taking them.” The participant further explained that if the doctor knew that she was not taking the medication, she would be involuntarily admitted to the hospital. The participant accepted her need to avoid weight gain even though it meant feeding into her eating disorder and being dishonest about taking the medication. *Meeting expectations* safeguarded her from pressures to prioritize the treatment of SI and losing control, a strategy that *made room to be OK* with the space to manage her psychological pain with agency.

Despite feeling physically and emotionally drained, women enacted their socially expected roles as caregivers despite limited capacity to do so. Not wanting to disappoint loved ones, a participant with a history of self-harming felt pressured to behave in ways that represented society’s views of being “strong.” She explained, “I’ve always had to be the one to have these discussions with my sister, because she self-harmed as well. So, I’ve always had to be strong [. . .] really exhausting.” *Meeting expectations* by prioritizing her mother and sister’s needs before her own was draining; however, doing so projected an image of being a strong caretaker which avoided letting her family down. Without others’ *acceptance* of their need to escape unbearable pain, lacking resources and supports from health services, and surrounded by gender expectations, women believed that they were not deserving of, had difficulty visualizing, and lacked the energy to use a different strategy to more effectively meet their health need to reduce psychological pain.

##### Permitting Myself to Be Me

In the context of low *compression*, *permitting myself to be me* occurs, a process of taking up space in the environment and allowing one’s authentic self to be seen and heard by resisting pressure to enact social expectations. In *permitting myself to be me*, internal *acceptance* is in full activation, with the assistance of external *acceptance*. It is here that women have the capacity to express their SI and psychological pain, free from expending energy to pretend that they are OK and have the mental space to determine what they need to do to feel better without guilt. Situated in structural *acceptance*, women find community or a sense of belonging where their uniqueness is valued. Higher socioeconomic status supported *permitting myself to be me*, providing women with access to organized communities of their choosing where they feel a sense of belonging and engagement. Access to post-secondary education wherein women engaged with others with similar ideology and values, employment with diverse co-workers, and financial access to therapy with a trusted counsellor provided spaces to explore oneself and to feel connected to others. A university student who felt isolated and lost during the COVID pandemic found a sense of purpose having intellectual conversations with people on campus, “I live for these discussions and having that academic community. And it was also really nice because really whenever I wanted, whenever I wanted to talk with a prof or my thesis supervisor, I could always just walk over to campus.”

*Permitting myself to be me* is resisting social pressure to change and resisting the pressure women feel to ignore their health needs to end psychological pain. Women let go of unrealistic goals and pressures to be productive when they have accepted their need for rest and a break from social responsibilities. A participant who had been feeling guilty about staying in bed on days when she experienced SI talked about how her friend’s *acceptance* of her health needs allowed her to rest. She recounted, “[I] legit just got out of bed, couldn’t bother to be productive, [my friend] said, ‘Sometimes you just need that!’ I stayed in bed all day on Sunday.” By not only resisting the social pressure to get out of bed, *acceptance* of her exhaustion and the need to pause her everyday responsibilities emboldened her to take an extended break by remaining in bed for the whole day.

*Permitting myself to be me* is evidence of a strong sense of intrapersonal *acceptance*, being highly aware of their personal identity, acknowledging the urgency of their health needs, and believing they are deserving of support and resources needed to feel a sense of belonging. Intrapersonal *acceptance* interrupts social pressures to please or to avoid bothering others, providing energy and focus to address their needs. After a long duration of SI, feeling depressed, and struggling to care for herself, one participant described coming to the realization that she was deserving of having “basic needs” like eating and sleeping met: “I need to make sure that I’m getting the healthcare that I need even though it’s frustrating and incredibly stressful, and gives me anxiety. I just have to keep saying to people, “I need this!” *Permitting myself to be me*, and prioritizing health needs as urgent and valid can be hard, but as noted in this exemplar, it is critical to *making room to be OK.*

In all, women with SI use a variety of different strategies to manage their health and well-being by *making room to be OK*. Which strategy employed depends on the level of *compression* and type of *acceptance* present.

## Discussion and Implications

*Making room to be OK*, a theoretical rendering of women’s management of their health in the context of SI, is in line with a novel way of constituting SI that exists in opposition to the dominant biomedical model. A strength-based approach to women’s health management and promotion while living with SI is a novel addition to the literature and clinical practice. Findings offer a shift in the narrative treating SI as a pathology toward understanding SI as a part of health. Deconstructing dominant conceptualizations of SI as pathology and its treatment as linear, findings challenge fear-based, crisis-orientated notions that SI must be controlled with paternalistic reactions. *Making room to be OK* broadens understanding of SI as a common and justifiable response to psychological pain that women know how to manage, by creating space and inviting opportunities to acknowledge and explore SI. Indeed, Shneidman (1993), a prominent suicidologist, wrote that SI is a human response to unmet psychological needs. A health and wellness lens to SI and other mental health problems among women calls for structural changes to how services are constructed and provided, and in how suicide and SI are conceptualized and researched.

### Shift to a Health Paradigm

Findings signal the need to reconceptualize how SI is approached. Situating SI within a health lens or as existing in parallel with possibilities for improved health contrasts with Liljedahl et al.’s (2023) recovery framework for women with borderline personality disorder, a linear process wherein health and wellbeing are achieved only after SI, self-harm, and other symptomology have abated. According to the *making room to be OK* theory, health is achievable regardless of SI intensity; dependent on each woman’s own definition or meaning of health, that they can *be OK* or confident in their capacity to manage psychological pain. This paradigm shift is grounded in an epistemological stance that values women’s knowledge, traditionally silenced or ignored in favour of dominant perceptions of health situated in medical interpretations, including ones that prioritize avoidance of death and self-harm. Paternalistic efforts to control how women think and pressure them to enact societal expectations is a rejection of women’s personhood; approaches that fail to acknowledge SI as a common and justifiable response to pain and trauma and intensifies women’s pain and SI (Taylor et al., 2025). Prioritizing autonomy and cultural safety increases access to support and services among women with SI who may be reluctant to seek help.

*Making room to be OK* acknowledges health as a subjective perception of quality of life that may be socially unacceptable because *being OK* may include SI and pausing social expectations to care for others. The finding that *making room to be OK is* contingent on *acceptance* calls for harm reduction approaches that integrate recognition of SI as a common response to psychological pain and calls for attention to managing health and psychological pain before mobilizing restrictive interventions intent on controlling self-harm behaviour. For example, listening to women’s SI in a way that normalizes the need to address psychological pain without conveying the need to change women’s thoughts and actions is supportive of women with SI. Mobilizing women’s strengths in managing pain, including SI, can be part of care planning, similar to how safe injection sites are used as harm reduction strategies for heavy substance use. Adjusting treatment goals to better align with *making room to be OK* is a strength-based approach that takes women’s needs and realities into account. *Making room to be OK* demonstrates that a broad range of health care providers ought not to prioritize the extinguishing of SI to help women promote their health but rather support them to *be OK* in the present, despite SI.

### Creating “Room”

Treatment interventions and structural changes are warranted to assist women to *make room* or spaces that are considered safe. Safe spaces, a term coined to describe anti-violent environments for LGTBQ2S+ communities where they will not be discriminated against based on their gender or sexuality (Zalman & McHenry-Sorber, 2023), aligns with study findings. The safety of having *room to be OK* is a space devoid of gendered attitudes that judge women with SI as being dramatic and attention seeking. However, *room to be OK* is different than “safe spaces” because it subsumes women’s active role in engaging in goal directed behaviour to manage pain. Whereas safe spaces are typically carved out as a particular physical environment such as an office or building where people congregate (Zalman & McHenry-Sorber, 2023), “*room*” exists beyond physical environments into an embodiment of time, energy, and space and can be created in any place at any time, alone or with others. The experience of a temporal lens in the management of SI is supported by a study that found people with SI experienced altered perceptions of how time was organized (Gay, 2025b). Vatankhah et al. (2024) found that attempted suicide was perceived as an embodiment of detachment from others that was experienced in relation to a lack of space and a desire for freedom, findings that align with the need for “*room*.”

Strategies to promote structural *acceptance* includes targeting barriers to *permitting myself to be me*, freedom within spaces that are reduced with statutory provisions for compulsory psychiatric care in many countries that permit involuntary hospital admissions, medical treatment, and other coercive practices including seclusion and restraint. Examining how mental health acts can be amended toward providing more ethical, equitable, anti-racist, and anti-ableist services (Kolar et al., 2023) will invite greater *acceptance* and *room to be OK*. Other needed changes to create structural *acceptance* are providing culturally safe environments that target social determinants of health. Financial support and changing cultural values that place women in a subordinate position in society were identified as protective against suicide and interpersonal violence among Muslim women (Alonzo & Zubaroglu-Ioannides, 2024) and are examples of structural acceptance that ought to be considered for women with SI.

Promoting interpersonal *acceptance* involves mobilizing safe interactions with health care providers in programs and services that demonstrate *acceptance* of SI, along with an *acceptance* of having difficulty functioning, and understanding that ending unbearable psychological pain is a health need. These efforts require understanding suicidality from a contextual standpoint, including considering the meaning women derive from SI and exploring one’s own comfort level in discussing self-harm and death. Baril (2020) calls for a “suicide-affirmation approach” (p. 217) where helpers use critical reflexivity to acknowledge paternalist interventions and instead offer environments in which people are free to discuss SI without fear of punishment in the form of judgmental attitudes, efforts to change their minds, and removing freedoms (Baril, 2020). Capacity for such environments within community and primary care settings may help to avoid waiting until SI intensifies, requiring crisis or tertiary services.

Attitudes and strategies that support women’s health promotion are strengthened by Rogers’ (1951) unconditional positive regard for the client that addresses relational power dynamics to improve self-worth (Kass, 2015). Unconditional positive regard may strengthen intrapersonal *acceptance*, helping women to acknowledge the difficulties related to SI and maintain a positive sense of self despite thinking of suicide, having difficulty functioning, and other characteristics that are socially unacceptable. Indeed, radical *acceptance*, a dialectical behavioural therapy inspired coping strategy that helps to let go of difficulties that are beyond one’s control, demonstrated effectiveness in regulating negative affect (Segal et al., 2025). The findings on *acceptance*, however, challenge prevailing frameworks in psychology, psychiatry, and related healthcare disciplines, which narrowly and exclusively construct SI and so-called “self-harm” as inherently maladaptive. The way SI can help women to *be OK* or manage pain is strengthening and adaptive, calling into question how dominant views of health and wellbeing contrast with those of women with SI.

### Limitations and Future Research

The study findings are limited to a mostly White sample. Given that non-White participants confronted racial barriers to *making room to be OK*, future research ought to explore these challenges in more depth. A Canadian sample (most from New Brunswick) may reduce generalizability of the findings due to health care supports that may differ in other countries. Data collection began during the COVID pandemic, a period of time where social restrictions prevented in person interviews. Although videoconferencing interviews yielded rich data, in-person meetings with participants may have created a greater sense of comfort and trust for the participants, promoting more in-depth discussion. Since suicidality is rarely explored with a health promotion lens, future research that explores what health means to women living with SI will provide greater insights into how suicidality fits with women’s overall health promotion and ways to support women to be well. A trauma and violence informed approach calls for an analysis on how structural and systemic factors influence women’s capacity to *make room to be OK*, including gendered expectations to care for others, stigma that women with SI are attention seeking, and how Western ideals of individualism, capitalism, and productivity act as barriers to addressing health needs for rest and psychological pain management.
